# Amperometric Hydrogen Peroxide Biosensor Based on Immobilization of Hemoglobin on a Glassy Carbon Electrode Modified with Fe_3_O_4_/Chitosan Core-Shell Microspheres

**DOI:** 10.3390/s90806185

**Published:** 2009-08-05

**Authors:** Xue-Cai Tan, Jin-Lei Zhang, Sheng-Wei Tan, Dan-Dan Zhao, Zen-Wei Huang, Yan Mi, Zai-Yin Huang

**Affiliations:** College of Chemistry and Ecological Engineering, Guangxi University for Nationalities, Nanning 530006, China; E-Mails: zhangjl1984123@yahoo.cn (J.-L.Z); PGtansw@126.com (S.W.T.); bczdd_198571@sina.com (D.-D.Z.); HZW8188516@126.com (Z.-W.H.); miyan9@163.com (Y.M.); hzy210@163.com (Z.-Y.H.)

**Keywords:** hemoglobin, hydrogen peroxide, magnetic microspheres, biosensor, hydroquinone

## Abstract

Novel magnetic Fe_3_O_4_/chitosan (CS) microspheres were prepared using magnetic Fe_3_O_4_ nanoparticles and the natural macromolecule chitosan. Then, using an easy and effective hemoglobin (Hb) immobilization method, an innovative biosensor with a Fe_3_O_4_/CS-Hb-Fe_3_O_4_/CS “sandwich” configuration was constructed. This biosensor had a fast (less than 10 s) response to H_2_O_2_ and excellent linear relationships were obtained in the concentration range of 5.0 × 10^−5^ to 1.8 × 10^−3^ M and 1.8 × 10^−3^ to 6.8 × 10^−3^ M with a detection limit of 4.0 × 10^−6^ M (s/n = 3) under the optimum conditions. The apparent Michaelis-Menten constant *K_m_* was 0.29 mM and it showed the excellent biological activity of the fixed Hb. Moreover, the biosensor had long-time stability and good reproducibility. The method was used to determine H_2_O_2_ concentration in real samples.

## 1. Introduction

Determination of hydrogen peroxide is of great interest because it is a by-product of several highly selective oxidases and it plays an important role in various fields such as food, pharmaceutical and environmental analysis [1–3]. Techniques for detecting H_2_O_2_ include titrimetry, chemiluminescence, spectrometry and electrochemical methods [4]. Among these techniques, electrochemical analyses have been extensively employed for determination of H_2_O_2_ because they offer intrinsic sensitivity, extended dynamic range and rapid response times [5].

Hemoglobin is a heme protein containing four electroactive iron hemes and it can store and transport oxygen in red blood cells [6]. It has commonly been employed to construct H_2_O_2_ biosensors as a result of its commercial availability and peroxidase activity [7]. However, the electron transfer reactivity of hemoglobin on conventional electrode surfaces is physiologically hampered, because its normal electroactive center is deeply buried in its electrochemically “insulated” peptide backbone [8]. Therefore many efforts have been made to enhance the electron transfer rate of hemoglobin by using mediators [9–11], promoters [12–14] and a variety of immobilization materials such as polymer films [15], surfactants [16], and nanomaterials [17–19].

In recent years, research has focused on magnetic nanoparticles. Due to their good biocompatibility, strong superparamagnetic properties, low toxicity and easy preparation processes, magnetic nanoparticles have been used in various fields, including enzyme immobilization [20–23]. As one of the most important materials, a biocompatible ferromagnetic material could interact with proteins via some active groups such as -OH, -COOH, -NH_2_, without any denaturation of absorbed proteins [24,25] and could serve as a modifier to promote electron transfer reactions [26], so there has been very intense research on this topic in the field of biosensors [27,28]. One of the main problems in the application of superparamagnetic nanoparticles is surface modification with various useful materials which can tailor them for specific applications in drug targeting, immobilization of enzymes, immunology, cell separation process and so on [29]. The polymer magnetic microsphere is one kind of useful magnetic carrier that can be synthesized by coating a layer of polymer film onto the magnetic nanoparticles. It has been successfully applied in enzyme immobilization for its abundant functional groups. Qiu *et al*. synthesized magnetic core-shell Fe_3_O_4_@SiO_2_ nanoparticles and constructed a glucose biosensor using amino-functionalized Fe_3_O_4_@SiO_2_ nanoparticles covalently bound with ferrocene monocarboxylic acid as the building block [30]. Lai *et al*. prepared a kind of magnetic microsphere and Hb could be immobilized on the surface of the microspheres by cross-linking with glutaraldehyde [31].

Chitosan (CS) has various desirable properties, e.g., biocompatibility, low toxicity, good film forming propertied, high mechanical strength and high hydrophilicity, so it has been a rather important material for preparation of magnetic carriers. Recently, chemically modified electrodes and enzymatic biosensors with chitosan have been reported for determination of Fe^3+^ [32], Pd^2+^ [33], H_2_O_2_ [34], glucose [35], cholesterol [36], etc. To the best of our knowledge, most previous research on magnetic microspheres in various fields, including enzyme immobilization, has focused on iron oxide as the magnetic core. However, few studies were reported the use of microspheres in biosensors. In this study, a type of magnetic microsphere was prepared by suspension crosslinking using Fe_3_O_4_ nanoparticles and chitosan. An amperometric hydrogen peroxide biosensor with a sandwich configuration was fabricated for the first time based on an immobilized Hb modified electrode and the mediator hydroquinone (HQ).

## 2. Experimental Section

### 2.1. Apparatus

Cyclic voltammetric and amperometric experiments and EIS investigations were performed with a CHI660A electrochemical workstation (Shanghai Chenhua Instrument Co., China). The three-electrode system consisted of a platinum wire counter electrode, Ag/AgCl (saturated KCl) reference electrode, and modified glassy carbon electrode (3.0 mm diameter) as working electrode. All the electrochemical processes were in aerated solutions. Transmission electron micrograph (TEM) images were obtained with a JEM-200CX (JEOL, Japan) instrument.

### 2.2. Materials

Hb from bovine blood was obtained from Fluka. Chitosan (CS, 95% deacetylation) was bought from Shanghai Biochemical (Shanghai, China). d-glucose and 30% H_2_O_2_ were obtained from Guangzhou Chemical (Guangzhou, China). Disinfectant was purchased from South Land Pharmaceutical. Other reagents were of analytical reagent grade. All solutions were prepared with doubly distilled water. HQ was purchased from Sinopharm Chemical Co. Ltd., China. The exact concentration of H_2_O_2_ was determined by titration against a standard potassium permanganate solution.

### 2.3. Synthesis of Fe_3_O_4_ Nanoparticles [37]

Fe_3_O_4_ nanoparticles were synthesized by co-precipitation of Fe^2+^ and Fe^3+^ ions in the presence of alkaline solution under hydrothermal treatment. 5.2 g of FeCl_3_·6H_2_O and 2.0 g of FeCl_2_·4H_2_O were dissolved in 25 mL of 0.4 M HCl. The solution of the mixed iron-salts was added dropwise into a solution of 250 mL 1.5 M NaOH with vigorous stirring under an atmosphere of nitrogen gas. Then the obtained black precipitate was heated at 75 °C for 30 min. The precipitate was collected through centrifugation at 4,000 rpm, washed sequentially with distilled water and ethanol. A black colored powder (Fe_3_O_4_ nanoparticles) was obtained upon drying under vacuum at 60 °C for 6 h.

### 2.4. Preparation of Magnetic Microspheres (Fe_3_O_4_/CS) [31]

The suspension cross-linking technique was used for the preparation of magnetic microspheres. A 20 mL 2.5% chitosan solution containing 0.4 g Fe_3_O_4_ nanoparticles was prepared using a 3% acetic acid solution. Then it was poured, dropwise, into the dispersion medium composing of 80 mL paraffine oil and 5 mL Span-80 (a type of nonionic surfactant). At the same time, the dispersion medium was stirred with a magnetic stirrer at 1,500 rpm at room temperature. After thirty minutes, 1.0 M NaOH was added slowly to the dispersion medium to adjust the mixture to pH 10.0 at the stirring rate of 1200 rpm. Fifteen minutes later, 0.4 mL of epoxychloropropane was added to the medium and reacted for 40 min at 50 °C. Similarly, an additional 0.4 mL of epoxychloropropane was added to the dispersion and the stirring rate was lowered to 1,000 rpm. After 1 h, the mixture was continuously reacted for a further 2 h with a lower stirring rate of 800 rpm at the temperature of 60–70 °C. At the end of this period, the magnetic microspheres were collected using a magnet and washed consecutively with ether, acetone, 10% ethanol and doubly distilled water, then vacuum dried at 50 °C prior to storage for further analysis and use.

### 2.5. Configuration of Fe_3_O_4_/CS-Hb-Fe_3_O_4_/CS-GCE

A glassy carbon electrode (GCE, 3 mm in diameter) was polished with 1.0, 0.3, and 0.05 μm Al_2_O_3_ slurry, and rinsed thoroughly with doubly distilled water in order to obtain a mirror-like surface. Then it was sonicated in 1:1 nitric acid, absolute ethanol and doubly distilled water for 5 min, respectively, and allowed to dry at room temperature. 2.0 mg Fe_3_O_4_/CS was dispersed into 1.0 mL doubly distilled water with 15 min ultrasonic agitation and 2.0 mg mL^−1^ Fe_3_O_4_/CS suspension was obtained. 5 μL of this suspension was cast onto the surface of GCE, and then it was dried in air. One hour later, a drop of Hb (8 mg mL^−1^) with the volume of 10 μL was cast onto the surface of the modified GCE. After drying in air for approximately 5 h, the electrode was then coated with 5 μL Fe_3_O_4_/CS dispersion and dried for about 1 h. Finally, 5 μL 8% glutaraldehyde was dropped. The resulting Fe_3_O_4_/CS-Hb-Fe_3_O_4_/CS-GCE was obtained after the modified electrode was dried in air for 1 h and immersed in a 0.15 M PBS (pH 8.0) for 1 h to remove the redundant adsorption of Hb.

## 3. Results and Discussion

### 3.1. TEM Characterization of Fe_3_O_4_ Nanoparticles and Magnetic Microspheres

**Figure 1.**
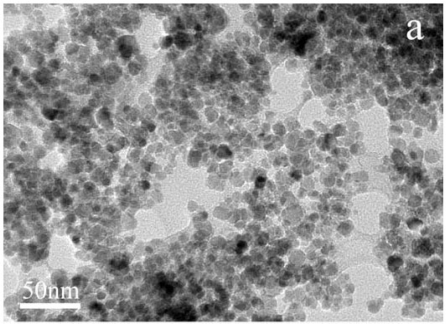

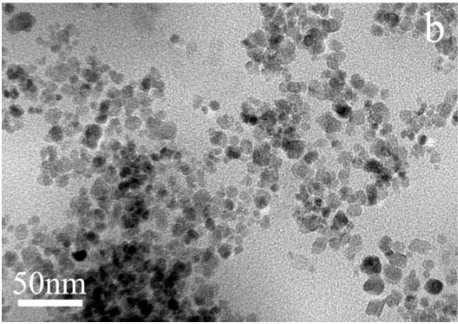
TEM photos of synthesized Fe_3_O_4_ nanoparticles (a) and magnetic microspheres (b).

Figure 1 displays the TEM images of Fe_3_O_4_ nanoparticles (a) and magnetic microspheres (b). The Fe_3_O_4_ particles are nanosized and the average size is approximate 10 nm, with uniform distribution. The average diameter of microspheres is about 20 nm, which is larger than that of Fe_3_O_4_ nanoparticles, showing that the chitosan has been coated on the surface of the Fe_3_O_4_ nanoparticles.

### 3.2. Optimization of Experimental Variables

**Figure 2.**
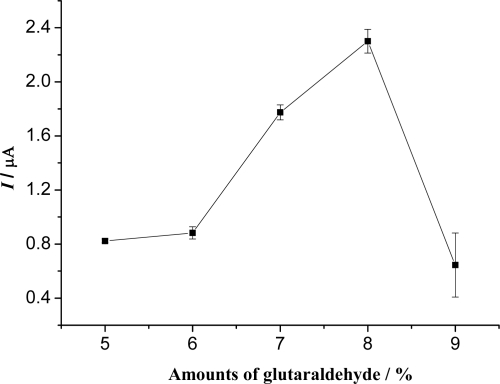
Effect of the glutaraldehyde concentration on the biosensor response to 5.0 × 10^−4^ M H_2_O_2_ in 0.15 M pH 8.0 PBS containing 6.0 × 10^−4^ M HQ.

To improve the performance of the biosensor, various factors influencing the response of the sensor such as amount of glutaraldehyde, the concentration of HQ, and the applied potential were investigated. Glutaraldehyde was used as a cross-linker in the construction of the biosensor. It plays an important role on the protein deposition, due to the cross-linking reaction between the amino groups of chitosan and the glyoxal groups of glutaraldehyde, and the glyoxal groups of glutaraldehyde and the amino groups of Hb. The effect of the concentration of glutaraldehyde used for the preparation of the biosensor is shown in Figure 2. It can be seen from Figure 2 that the biosensor prepared with 8% glutaraldehyde solution had a maximum response. The reason may be that: if the concentration of glutaraldehyde is low, the amount of immobilized protein is low and the response of the biosensor is low. However, if the concentration of glutaraldehyde is higher, the immobilization film is thicker, leading to a higher diffusion barrier. So 8% glutaraldehyde was selected as the cross linker.

**Figure 3.**
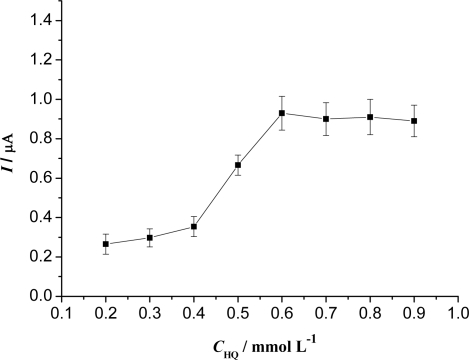
Effect of HQ concentration on the biosensor response to 5.0 × 10^−4^ M H_2_O_2_ in 0.15 M pH 8.0 PBS.

As shown in Figure 3, the response of the modified electrode increased gradually as the HQ concentration was increased, reaching a maximal value of 6.0 × 10^−4^ M, and then the current did not change with further increases of the HQ concentration. Such a behavior is typical of a mediator-based sensor [38,39]. At a low HQ concentration, the current response is limited by enzyme-mediator kinetics. On the other hand, when the HQ concentration is too high, it results in a response limited by enzyme-substrate kinetics. However, a higher concentration of mediator produced a larger background current. Thus, 6.0 × 10^−4^ M HQ was chosen in the subsequent experiments.

**Figure 4.**
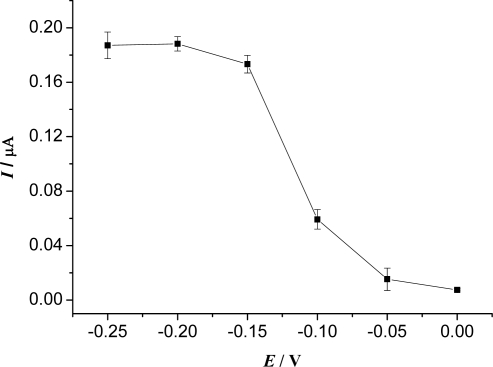
Effect of the working potential on the biosensor response for 5.0 × 10^−4^ M H_2_O_2_ in 0.15 M pH 8.0 PBS containing 6.0 × 10^−4^ M HQ.

Figure 4 shows the effect of the working potential on the steady-state current of the biosensor in the potential range from 0 to −0.25 V in pH 8.0 PBS containing 6.0 × 10^−4^ M HQ in the presence of 5.0 × 10^−4^ M H_2_O_2_. The steady-state current of electrocatalytic reduction of H_2_O_2_ increased gradually when the applied potential shifted from 0 to −0.15 V and then increased slowly as the potential became more negative from −0.15 to −0.25 V. Although a higher signal current was achieved at −0.25 V, the background current also increased distinctly. Moreover, lower potential can bring interferences from electroactive species. Therefore, −0.15 V was selected as the working potential for the amperometric measurement of H_2_O_2_.

### 3.3. Electrochemical Characteristics of the Biosensor

**Figure 5.**
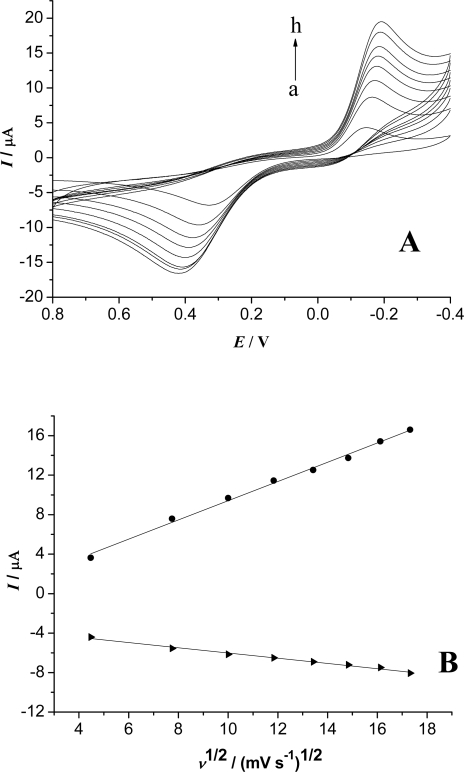
The cyclic voltammograms of Fe_3_O_4_/CS-Hb-Fe_3_O_4_/CS-GCE with different scan rates (from a to h: 20, 60, 100, 140, 180, 220, 260, 300 mV/s) in 0.15 M pH 8.0 PBS containing 6.0 × 10^−4^ M HQ (A). (B) the linear relation between peak current and *v*^1/2^.

Figure 5A shows the cyclic voltammograms of the Fe_3_O_4_/CS-Hb-Fe_3_O_4_/CS-GCE in 0.15 M pH 8.0 PBS containing 6.0 × 10^−4^ M HQ with different scan rates. Both anodic and cathodic peak currents increased linearly with the square root of scan rate (Figure 5B). From the results, we can confirm that a diffusion-controlled process occurred at the Fe_3_O_4_/CS-Hb-Fe_3_O_4_/CS-GCE. The result was consistent with the previous report [31].

**Figure 6.**
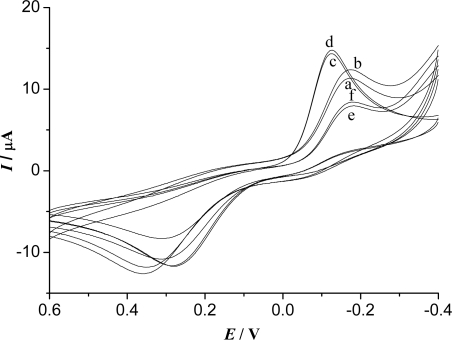
Cyclic voltammograms of Fe_3_O_4_/CS-Hb-Fe_3_O_4_/CS-GCE (a,b), Fe_3_O_4_/CS-GCE (c,d) and Fe_3_O_4_/CS-Hb-GCE (e,f) in the absence (a,c,e) and in the presence (b,d,f) of 5.0 × 10^−4^ M H_2_O_2_ in 0.15 M pH 8.0 PBS containing 6.0 × 10^−4^ M HQ, scan rate: 100 mV/s.

Figure 6 shows the cyclic voltammograms of the different modified electrodes in the absence and presence of H_2_O_2_. As seen in Figure 6a and b, when 5.0 × 10^−4^ M H_2_O_2_ was added, the reduction current increased and the oxidation current decreased significantly at the Fe_3_O_4_/CS-Hb-Fe_3_O_4_/CS-GCE, indicating that an obviously catalytic reduction current of H_2_O_2_ was caused at the biosensor. However, hardly any clear current response to H_2_O_2_ can be observed at the Fe_3_O_4_/CS-GCE (c and d). The results demonstrated that the current response of the biosensor to H_2_O_2_ was mainly due to the catalytic reduction effect of Hb and the immobilized Hb remained its activity. The reason may be that the Fe_3_O_4_/CS can effectively absorb Hb and provide an excellent biocompatible environment on the electrode surface. The response to H_2_O_2_ of the Fe_3_O_4_/CS-Hb-Fe_3_O_4_/CS-GCE (a and b) was obviously larger than that of the Fe_3_O_4_/CS-Hb-GCE (e and f), suggesting that the “sandwich” configuration could more effectively promote the electron transfer. The reaction mechanism of the biosensor can be summarized as follows:

$$\begin{array}{l} H_{2}O_{2}+\text{Hb}\;(\text{red})\to H_{2}O+\text{Hb}(O)\;(\text{Ox})\;..\\ \text{Hb}(O)\;(\text{Ox})+\text{HQ}\;(\text{Red})\to \text{Hb}\;(\text{red})+\text{Q}\;(\text{Ox})+H_{2}O\\ \text{Q}\;(\text{Ox})+{2e}^{-}+2H^{+}\to \text{HQ}\;(\text{Red}) \end{array}$$

And the net reaction is H_2_O_2_ + 2e^−^ + 2H^+^ → 2H_2_O.

### 3.4. Electrochemical Impedance Spectroscopy Characterization

**Figure 7.**
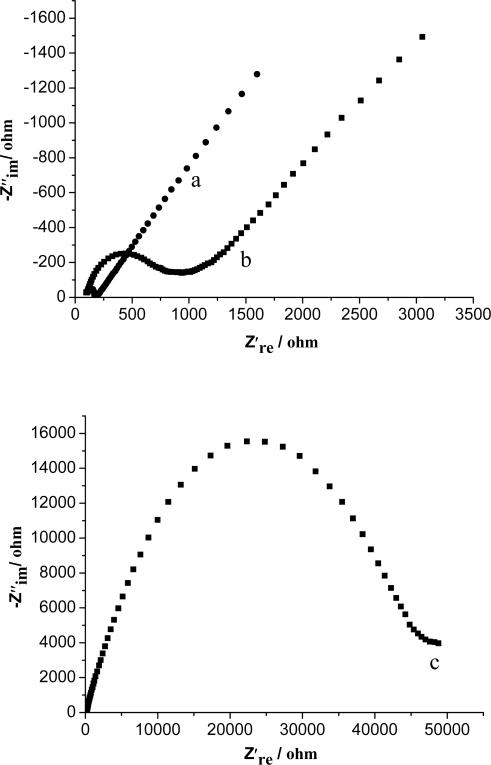
Electrochemical impedance spectroscopy of different modified electrodes: the bare GCE (a), the Fe_3_O_4_/CS-GCE (b), the Fe_3_O_4_/CS-Hb-Fe_3_O_4_/CS-GCE (c) in 5.0 × 10^−3^ M K_3_Fe(CN)_6_/K_4_Fe(CN)_6_ (1:1) containing 0.1 M KCl.

Electrochemical impedance spectroscopy (EIS) can provide useful information on the impedance changes of the electrode surface during the fabrication process. The Nyquist plot of the EIS includes a semicircular portion and a linear portion. The semicircle part at higher frequencies corresponds to the electron-transfer limited process. Its diameter is equal to the electron transfer kinetics resistance, *R*_et_, which controls the electron transfer kinetics of the redox probe at the electrode interface [40]. Figure 7 displays the Nyquist plots of the EIS of bare GCE (a), Fe_3_O_4_/CS-GCE (b), and Fe_3_O_4_/CS-Hb-Fe_3_O_4_/CS-GCE (c) in 5.0 × 10^−3^ M K_3_Fe(CN)_6_/K_4_Fe(CN)_6_(1:1) containing 0.1 M KCl. The Nyquist diameter of the Fe_3_O_4_/CS-GCE is much larger than that of the bare GCE, which suggests that Fe_3_O_4_/CS coated on the electrode can block the electron transfer of the redox probe. Compared with the Fe_3_O_4_/CS-GCE, an obvious increase in the interfacial resistance is observed at the Fe_3_O_4_/CS-Hb-Fe_3_O_4_/CS-GCE, which indicated that Hb was immobilized successfully on the electrode. The Nyquist diameter of the Fe_3_O_4_/CS-GCE is much smaller than that of the MCMS/GCE [31], showing that Fe_3_O_4_ nanoparticles have more excellent conductivity than carbon-coated iron nanoparticles. However, the *R*_et_ of the Fe_3_O_4_/CS-Hb-Fe_3_O_4_/CS-GCE is obviously larger than that of Hb/MCMS/GCE [31]. This may be ascribed to the larger surface area of Fe_3_O_4_/CS and the specificity of the “sandwich” configuration, leading to more Hb immobilized onto the Fe_3_O_4_/CS-Hb-Fe_3_O_4_/CS-GCE.

### 3.5. Amperometric Response of the H_2_O_2_ Biosensor

**Figure 8.**
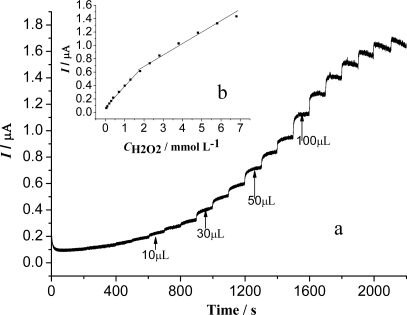
Amperometric response of the biosensor to successive additions of H_2_O_2_ in 0.15 M PBS containing 6.0 × 10^−4^ M HQ. The working potential was −0.15 V. (inset) a plot of amperometric response vs, H_2_O_2_ concentration.

The typical steady-state amperometric response of the biosensor was investigated by successively increasing the H_2_O_2_ concentration under the optimized conditions. Figure 8 shows the chronoamperometric curve of the biosensor for successive addition of H_2_O_2_ in a magnetic stirring condition. When the H_2_O_2_ was added into the pH 8.0 PBS containing 6.0 × 10^−4^ M HQ, the biosensor responded rapidly to the substrate increase and achieved 95% of the steady current in 10 s. It is faster than the reported results of HRP that was immobilized on a hydrophilic matrix film modified glassy carbon electrode (40 s) [41]. This may result from the Fe_3_O_4_ nanoparticles that can facilitate the transfer of electrons. The inset shows the calibration curve between amperometric current and H_2_O_2_ concentration. Excellent linear relationships were obtained in the concentration range of 5.0 × 10^−5^–1.8 × 10^−3^ M and 1.8 × 10^−3^–6.8 × 10^−3^ M. The regression equations were expressed as *i*_p_ (μA) = 0.07077 + 0.3150*c* (mM), R = 0.997 and *i*_p_ (μA) = 0.3673 + 0.1340*c* (mM), R = 0.993. The detection limit of the biosensor was estimated as 4.0 × 10^−6^ M at a signal to noise ratio of 3. The linear ranger is broader than that of the Hb/MCMS/GCE (MCMS were prepared using carbon-coated iron nanoparticles as the magnetic core) [31]. The result indicates that the magnetic microspheres in the experiment displayed better catalytic ability than the microspheres using the magnetic core of carbon-coated iron nanoparticles.

The apparent Michaelis-Menten constant (*K*_m_^app^), which reflects the enzymatic affinity and the ratio of microscopic kinetic constants, can be obtained from electrochemical version of the Lineweaver-Burk equation [42]. *K*_m_^app^ is calculated to be 0.29 mM, which is smaller than that those of 0.68 mM for Hb in hydrophilic matrix film modified electrode [41], 0.75 mM for Hb in chitosan/nano-CaCO_3_ composite electrode [43], and 0.898 mM for Hb in silica sol-gel film modified electrode [44]. These results suggest that the immobilized Hb remains its activity to H_2_O_2_ reduction and the biosensor has high affinity to H_2_O_2_.

### 3.6. Interference and Selectivity

Interference experiments for the biosensor were performed by comparing the amperometric responses to 5.0 × 10^−4^ M H_2_O_2_ before and after addition of possible interferents to 0.15 M pH 8.0 PBS containing 6.0 × 10^−4^ M HQ. The results from the test are listed in Table 1. In the experiments, 1.0 × 10^−3^ M glucose, l-cystine, l-lysine, l-glycine and uric acid did not cause any observable interference. However, an obvious interference was observed when 5.0 × 10^−3^ M ascorbic acid was added. The reason may be that the electroactive ascorbic acid can be oxidized by the HQ mediator. So the oxidation form of the mediator can be reduced and the cyclic catalytic reaction is destroyed [45].

### 3.7. Biosensor Repeatability and Stability

The repeatability of the biosensor was examined using the same electrode in the presence of 5.0 × 10^−4^ M H_2_O_2_. The relative standard deviation (RSD) was 4.6% for five successive assays. The fabrication repeatability of five Hb electrodes, prepared independently, shows an acceptable reproducibility, with a RSD of 4.8% for the response to the same concentration of H_2_O_2_. The biosensor was stored in a pH 8.0 PBS at 4 °C and measured intermittently (every 2 days). After 30 days, the biosensor retained about 89.5% of its original response. The stability of the biosensor is much better than that of previous biosensor [39]. Good long-term stability can be attributed to the sandwich configuration, which can more effectively immobilize Hb.

### 3.8. Analytical Application of the Biosensor

The accuracy of the biosensor was evaluated by determining the recoveries of hydrogen peroxide in a disinfectant using a standard addition method. Corresponding experiments were carried out with the titration method and the results displayed good consistent and precision between the two methods, as listed in Table 2. As seen in Table 2, the results obtained by the biosensor are satisfactory, with the recovery ranging from 98.1% to 107.7%.

## 4. Conclusions

Recently there has been increasing interest in in surface modification of magnetic nanoparticles with various useful materials. In this article, we introduced an amperometric H_2_O_2_ biosensor based on a Hb and Fe_3_O_4_/CSa “sandwich” configuration. The abundant amino groups present in Fe_3_O_4_/CS provide a biocompatible environment for Hb immobilization and glutaraldehyde acts as the cross linker. The biosensor exhibited a fast response to H_2_O_2_, and excellent linear relationships were obtained in the concentration range from 5.0 × 10^−5^ to 1.8 × 10^−3^ M and 1.8 × 10^−3^ to 6.8 × 10^−3^ M under the optimized experimental conditions, with a detection limit of 4.0 × 10^−6^ M (s/n = 3). The selectivity, repeatability and stability of the biosensor were also investigated, with satisfactory results, so this method could be used to construct a H_2_O_2_ biosensor for a variety of applications.

## Figures and Tables

**Table 1.** Determination results of possible interferences tested with the biosensor.

**Possible interferences**	**Current ratio^a^**

Glucose	1.02
l-Cystine	0.99
l-Lysine	1.01
l-Glycine	1.01
Uric acid	1.06
Ascorbic acid	0.37

a Ratio is the current for a mixture of interfering substance and 5.0 × 10^−4^ M H_2_O_2_ versus that for 5.0 × 10^−4^ M H_2_O_2_ alone. Other conditions as in Figure 6.

**Table 2.** Recovery determination results.

**Sample number**	**Added H_2_O_2_ (mM)**	**Found^a^ H_2_O_2_ by the present biosensor (mM)**	**Recovery by the resent biosensor (%)**	**Found^a^ H_2_O_2_ by the titration method (mM)**	**Recovery by the titration method (%)**

1	0.2	0.846	100.5	0.837	96.0
2	0.6	1.291	107.7	1.283	106.3
3	1.2	1.822	98.1	1.850	100.4

a The average of five measurements.
